# Alpine glacier algal bloom during a record melt year

**DOI:** 10.3389/fmicb.2024.1356376

**Published:** 2024-02-20

**Authors:** Jasmin L. Millar, Emily L. M. Broadwell, Madeleine Lewis, Alexander M. C. Bowles, Andrew J. Tedstone, Christopher J. Williamson

**Affiliations:** ^1^Bristol Glaciology Centre, School of Geographical Sciences, University of Bristol, Bristol, United Kingdom; ^2^British Antarctic Survey, Cambridge, United Kingdom; ^3^Department of Geosciences, University of Fribourg, Fribourg, Switzerland

**Keywords:** glacier algae, streptophyte, Morteratsch, glacier melt, Alps, climate change

## Abstract

Glacier algal blooms dominate the surfaces of glaciers and ice sheets during summer melt seasons, with larger blooms anticipated in years that experience the greatest melt. Here, we characterize the glacier algal bloom proliferating on Morteratsch glacier, Switzerland, during the record 2022 melt season, when the Swiss Alps lost three times more ice than the decadal average. Glacier algal cellular abundance (cells ml^−1^), biovolume (μm^3^ cell^−1^), photophysiology (F_v_/F_m_, rETR_max_), and stoichiometry (C:N ratios) were constrained across three elevations on Morteratsch glacier during late August 2022 and compared with measurements of aqueous geochemistry and outputs of nutrient spiking experiments. While a substantial glacier algal bloom was apparent during summer 2022, abundances ranged from 1.78 × 10^4^ to 8.95 × 10^5^ cells ml^−1^ of meltwater and did not scale linearly with the magnitude of the 2022 melt season. Instead, spatiotemporal heterogeneity in algal distribution across Morteratsch glacier leads us to propose melt-water-redistribution of (larger) glacier algal cells down-glacier and presumptive export of cells from the system as an important mechanism to set overall bloom carrying capacity on steep valley glaciers during high melt years. Despite the paradox of abundant glacier algae within seemingly oligotrophic surface ice, we found no evidence for inorganic nutrient limitation as an important bottom-up control within our study site, supporting our hypothesis above. Fundamental physical constraints may thus cap bloom carrying-capacities on valley glaciers as 21st century melting continues.

## Introduction

Streptophyte glacier algae (Procházková et al., 2021) represent the dominant primary producers in glacier surface ice environments, forming large-scale blooms during summer melt seasons that can approach densities of 10^3^-10^5^ cells per ml of meltwater under optimal growth conditions (Yallop et al., 2012; Williamson et al., 2018; Di Mauro et al., 2020; Irvine-Fynn et al., 2021). At these densities, glacier algal blooms lend a distinctive brownish/purple colouration to the ice surface because of the cells' heavy investment in secondary phenolic pigmentation (Remias et al., 2012; Williamson et al., 2020); shown to be a key adaptation to life in surface ice that protects against high irradiance (Williamson et al., 2020). Given the substantial ice surface darkening and resulting albedo decline associated with the growth of these heavily pigmented microalgal cells (Yallop et al., 2012; Di Mauro et al., 2020; Halbach et al., 2022), glacial algal blooms hold significant potential to exacerbate the melting of glacial ice across the cryosphere: in Greenland, the presence of algae was found to increase melting of high biomass regions by as much as 26% (Cook et al., 2020). As liquid water is considered a pre-requisite for glacier algal growth within ice (Williamson et al., 2019), the potential exists for establishment of a positive feedback loop between glacier algal growth, ice surface darkening, liquid water generation, and continued bloom proliferation. Accordingly, larger blooms are anticipated in years that experience the greatest overall melt (Tedstone et al., 2017; Williamson et al., 2019; Cook et al., 2020; Di Mauro et al., 2020).

Record melt years have recently been witnessed across the cryosphere, with the European Alps experiencing a record amount of glacier melt during the summer of 2022, when 5.9% of the total ice volume was lost (World Glacier Monitoring Service, 2022). In the Ötztal Alps of Austria, for example, the winter mass balance of Hintereisferner glacier was 43% below the decadal average of 2011–2020, paving the way for an extreme glacial mass loss during the summer season (Voordendag et al., 2023). Melting started earlier than normal, with the first positive degree days occurring in early May, initiating a nearly uninterrupted melt season that resulted in loss of 3,319 kg of ice per m^−2^, 3.4–5 times greater than the two proceeding years and 3.3 times greater than the 1991–2020 average (Voordendag et al., 2023). In the Swiss Alps, the average storage change for all Swiss glaciers over the 2022 melt season was estimated at 3.63 ± 0.26 km^3^ water equivalent, roughly 60% greater (three times higher) than the average annual loss over the past decade (Cremona et al., 2023). This was similarly driven by exceptionally low winter accumulation and high summer melting, the latter caused by a record number of 23 extreme heat wave events (defined as at least three consecutive days with a daily average maximum temperature >30°C) that occurred during summer 2022 (GLAMOS, 2022; Cremona et al., 2023). The consequences of these rapid melt events have a multitude of impacts on the surrounding environment and present many forms of threat to the safety of local communities (Beniston et al., 2011; Farinotti et al., 2023; Trautmann et al., 2023).

Within this context, 2022 was an important year to study glacier algal blooms, allowing to examine whether expected outcomes in bloom magnitude were apparent given the extreme melt conditions experienced. To this end, we report on a glacier algal bloom on Morteratsch glacier, Switzerland, during the 2022 summer melt season. Morteratsch glacier is the largest in the Bernina Range of Switzerland and is known to host extensive glacier algal blooms during most summer seasons (Di Mauro et al., 2017, 2020). Previous studies have identified a long-term darkening trend of surface ice at Morteratsch glacier (Oerlemans et al., 2009; Wientjes et al., 2011), with subsequent studies highlighting the role of glacier algae in albedo decline at this location (Di Mauro et al., 2017, 2020). The presence of both dominant glacier algal species, *Ancylonema nordenskiöldii* and *A. alaskana*, is known at Morteratsch glacier, with abundances reported at ~10^4^ cells ml^−1^ during the 2016 melt season (Di Mauro et al., 2020). Here, we provide a characterization of the glacier algal bloom at Morteratsch glacier during August 2022 to examine whether record melt conditions resulted in record bloom proliferation within this location.

## Methods

### Field site and sampling locations

**Figure 1:**
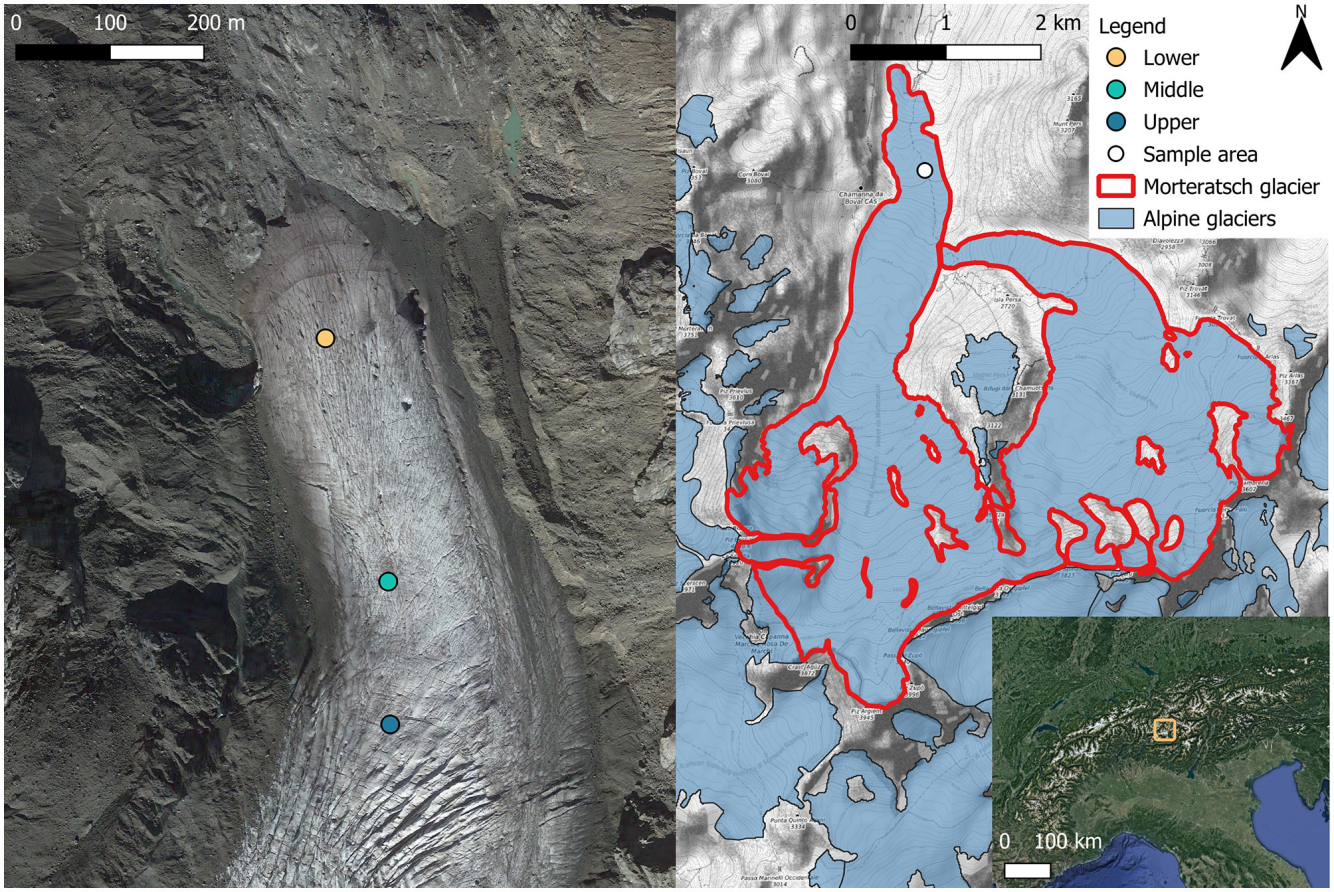
Morteratsch Glacier near Pontresina, Switzerland, showing glacier algae sampling area locations. Areas were chosen to cover the end ablation area which was populated by the glacier algal bloom. In total, 30 samples were taken from each location area (lower, middle, and upper) across 3 time points (morning, afternoon, and evening) over the 21st−23rd August 2022 for assessment of algae abundance, species composition, and local geochemistry. The bottom right map shows the location of Morteratsch in the European Alpine region. Base maps: Copernicus © 2022, satellite image taken October 2022.

**Figure 2:**
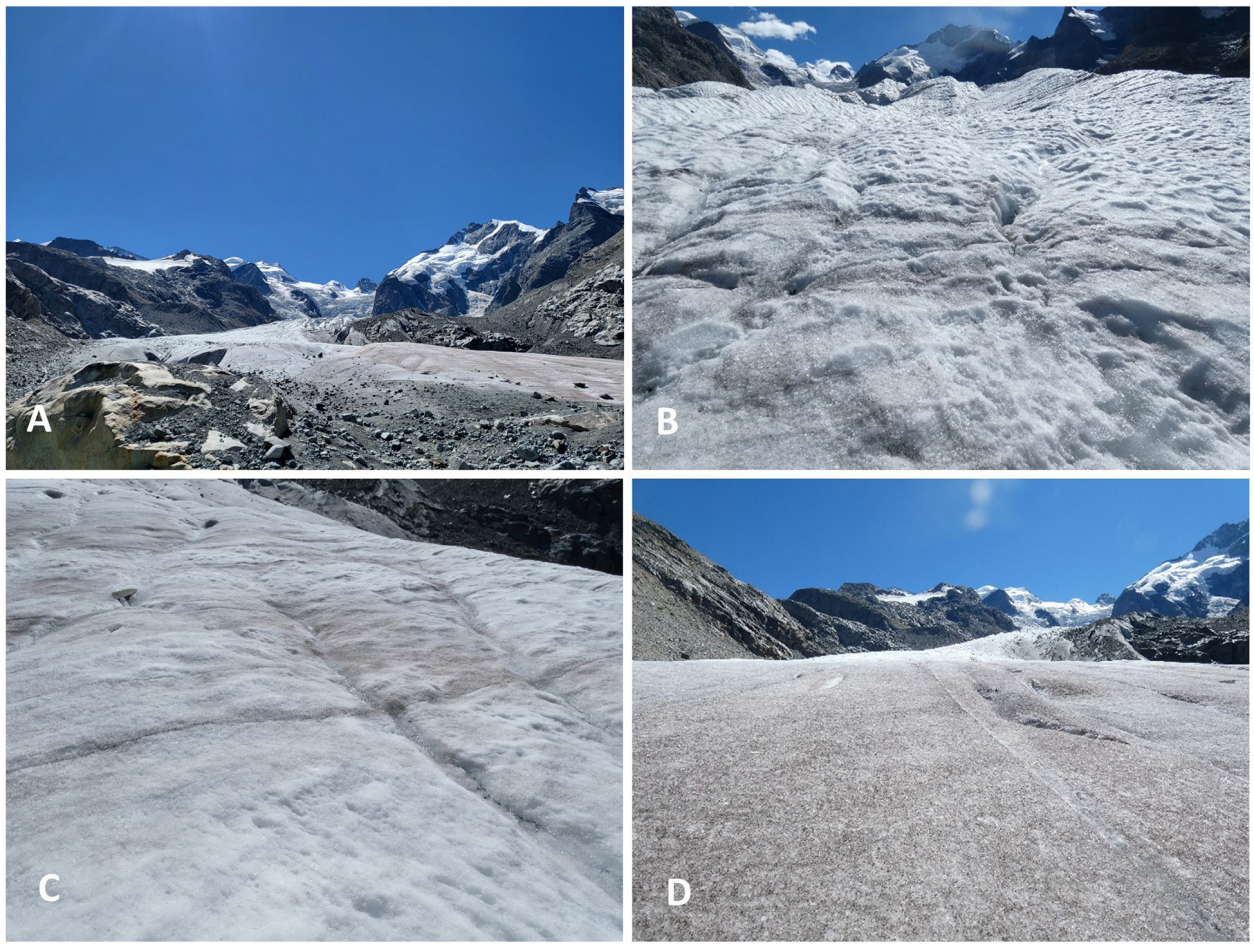
Photos of sampling areas on Morteratsch glacier, Switzerland. **(A)** View looking up Morteratsch glacier from the terminal moraine. The brown/purple color present toward the lower part of the glacier is indicative of a dense glacier algae bloom. **(B)** View of the “upper” sampling site (2,300 m elevation). **(C)** View of the “middle” sampling site (2,250 m elevation). **(D)** View of the “lower” sampling site (2,200 m elevation).

Characterization of the glacier algal bloom proliferating on surface ice of Morteratsch glacier, Switzerland, was undertaken over a 3-day period in late August 2022 (21st August 2022–23rd August 2022). Surface ice samples were collected across three main sites (one site sampled per day) distributed from the terminus of the glacier at 2,100 m up to 2,300 m elevation above sea level (Figure 1), hereafter referred to as lower (46.418N, 9.933E; Figure 2D), middle (46.415N, 9.934E; Figure 2C), and upper (46.414N, 9.934E; Figure 2B) sampling locations. At each location, *n* = 10 randomly selected areas of supraglacial surface ice were sampled at the start (10:00–12:00), middle (14:00–16:00), and end (17:00–19:00) of the day to capture potential diurnal dynamism in the biogeochemistry associated with blooms, with *n* = 30 samples per lower/middle/upper sampling location. All sampling was undertaken during clear, sunny-sky conditions (Figure 2). For each individual ice sample a 20 × 20 × 2 cm depth patch of surface ice was sampled using an ice saw and metal scoop, pre-sterilised with 10% ethanol, directly into a sterile Whirl-Pak bag using standard sampling techniques (Williamson et al., 2018, 2020). Before collection of each ice sample, an image with a scale was taken, and the slope, aspect, elevation with respect to sea level, and GPS coordinates were recorded (directly adjacent to sampling patch to avoid contamination) using the Phyphox app on an Apple iPhone 14 (Staacks et al., 2018).

### Sample processing

Ice samples were melted in the dark over 1–2 days at 4°C and homogenized prior to further processing. An initial 1.5 ml of each homogenized sample was placed into an individual 1.5 ml Eppendorf tube and fixed using Lugol's solution for subsequent cell counts and biovolume estimates at the University of Bristol, UK. A known volume of sample was then filtered through a pre-combusted (450°C for 5 h) 13-mm diameter GF/A filter (1.6 μm retention; Cytiva Whatman, Maidstone, UK), which was placed into an individual 1.5 ml Eppendorf tube and maintained at −20°C for subsequent cellular stoichiometry determination. Filtrates were collected into pre-acid-washed high density polyethylene (HDPE) bottles and maintained at −20°C for subsequent inorganic (ammonium, nitrate, nitrite, and phosphate) and organic (total organic carbon and nitrogen) aqueous geochemistry quantification. Samples were then driven to the University of Bristol, UK, and maintained within portable fridges at 4°C or freezers at −20°C.

### Nutrient addition experiments

To examine potential bottom-up control of glacier algal blooms at Morteratsch glacier, a nutrient spiking experiment was conducted *ex situ* with glacier algal communities sampled from surface ice. Fifteen samples were collected as above from lower Morteratsch glacier (46.418N, 9.933E) on 26th August 2022 and transported in the dark at 1°C within a portable fridge to Photon Systems Instruments, Czech Republic, where they were melted over 2 days as above. Approximately 7 days after sample collection, an incubation experiment was established with melted surface ice samples and their constituent glacier algal communities. Melted samples were initially pooled and then 50 ml distributed into *n* = 5 replicates across each of five treatments: control (no nutrient addition), +ammonium (+${\text{NH}}_{4}^{+}$, 10 μmol L^−1^), +nitrate (+${\text{NO}}_{3}^{-}$, 10 μmol L^−1^), +phosphate (+${\text{PO}}_{4}^{3-}$, 1 μmol L^−1^), and +ALL (+${\text{NH}}_{4}^{+}$ and +${\text{NO}}_{3}^{-}$ at 10 μmol L^−1^ and +${\text{PO}}_{4}^{3-}$ at 2 μmol L^−1^). ${\text{NH}}_{4}^{+}$, ${\text{NO}}_{3}^{-}$, and ${\text{PO}}_{4}^{3-}$ spikes were added as ${\text{NH}}_{4}^{+}$-N, NO_3_-N, and ${\text{PO}}_{4}^{3-}$-P standards, respectively (Thermo Fisher Scientific, UK). Treatments were chosen to provide ~10 times the reported ambient inorganic nitrogen and phosphorus concentrations in supraglacial ice (Wadham et al., 2016; Smith et al., 2017) and maintain a 10:1 nitrogen:phosphorus ratio within our “+ALL” treatment (McCutcheon et al., 2021). Incubations were conducted within 50 ml Corning culture flasks with vented caps (Corning, New York, USA) and maintained at 4°C under 500 μmol photons m^−2^ s^−1^ of light on a 16:8 Light:Dark cycle provided by warm white fluorescent tubes with no UV provision. Destructive sampling of *n* = 5 replicate incubations per treatment proceeded at 0, 24, 72, and 120 h for the determination of cellular abundance/biovolume, algal photophysiology, and cellular stoichiometry as described below.

### Abundance and biovolume determination

The cellular abundance (cells ml^−1^) and average biovolume per species (μm^3^ cell^−1^) were assessed for all samples collected in the field and used for incubation experiments. Cellular abundance was measured by counting cells on a modified Fuchs Rosenthal Hemocytometer (0.2 mm by 1/16 mm^2^; Hawksley, Lancing, United Kingdom) using a bright field Olympus BX41 microscope (Germany). Images of each sample were taken at 10 × and 40 × magnification with a MicroPublisher 6 CCD camera attachment (Teledyne Photometrics, USA), from which cells were counted and the width and radius measured for each sample using ImageJ software (Schneider et al., 2012). Final cellular biovolumes were calculated assuming species to be an average cylinder (Hillebrand et al., 1999). Power analysis was applied to determine the number of cells to measure to achieve a margin of error < 250 μm^3^ per species/sample (Jones et al., 2003). The total algal biovolume per sample (μm^3^ ml^−1^) was calculated by multiplying species' cellular abundances per sample (cells ml^−1^) by their corresponding mean biovolumes (μm^3^ cell^−1^).

### Photophysiology determination

Rapid light response curves (RLCs; Perkins et al., 2006) were performed using Pulse Amplitude Modulation (PAM) fluorometry to characterize the photophysiological states of glacier algal communities as sampled from Morteratsch glacier and throughout nutrient spiking experiments. Measurements were conducted on 3 ml subsamples using a Walz Water-PAM fluorometer with attached red-light emitter/detector cuvette system and stirrer (Walz GmBH, Germany). Each sample was dark adapted for a minimum of 5 min prior to RLC measurement. RLCs consisted of nine sequential light steps of 20 s duration ranging in irradiance from 0 to 3,000 μmol photons m^−2^ s^−1^ of Photosynthetically Active Radiation (PAR). The maximum quantum efficiency (*F*_*v*_*/F*_*m*_) was calculated from minimum (*F*_0_) and maximum (*F*_*m*_) fluorescence yields measured in the dark-adapted state during the initial RLC step of 20 s darkness as *Fv/Fm* = *Fm* – *Fo*/*Fm* (Consalvey et al., 2005). Electron transport through photosystem II (PSII) was calculated across subsequent light steps in relative units (*rETR*) assuming an equal division of light between PSI and PSII as rETR = Y (PSII) × PAR × 0.5 (Consalvey et al., 2005). Analysis of all RLC data (rETR vs. PAR) followed Eilers and Peeters (1988) for calculation of the relative maximum electron transport rate (rETRmax).

### Aqueous geochemistry determination

Aqueous concentrations of ${\text{NH}}_{4}^{+}$, ${\text{NO}}_{3}^{+}$, ${\text{NO}}_{2}^{-}$, and ${\text{PO}}_{4}^{3-}$ were derived for all melted ice samples spectrophotometrically using a Gallery Plus Discrete Photometric Analyser (Thermo Fisher Scientific, UK). The limit of detection (LoD) for all nutrients was determined by the mean concentration plus three times the standard deviation of calibration blanks (*n* = 3). LoDs were 0.04 μmol L^−1^ (${\text{NH}}_{4}^{+}$), 0.02 μmol L^−1^ (${\text{NO}}_{2}^{-}$), 0.15 μmol L^−1^ −(-${\text{NO}}_{3}^{-}$), and 0.15 μmol L^−1^ (${\text{PO}}_{4}^{3-}$). Precisions were ±2.0% (${\text{NH}}_{4}^{+}$), ±1.0% (NO_2_), ±1.3% (${\text{NO}}_{3}^{-}$), and ±2.0% (${\text{PO}}_{4}^{3-}$) as determined by comparison with diluted 71.43 mmol L^−1^ ${\text{NH}}_{4}^{+}$-N, ${\text{NO}}_{2}^{-}$-N, and ${\text{NO}}_{3}^{-}$-N and diluted 32.29 mmol L^−1^ ${\text{PO}}_{4}^{3-}$-P certified stock standards to a concentration of 3.6 μmol L^−1^ (${\text{NH}}_{4}^{+}$ and ${\text{NO}}_{2}^{-}$), 2.9 μmol L^−1^ (${\text{NO}}_{3}^{-}$), and 6.1 μmol L^−1^ (${\text{PO}}_{4}^{3-}$) (Sigma TraceCERT^®^).

Filtrate was also analyzed for total organic carbon (TOC) and total nitrogen (TN) concentrations via a TOC/TN Organic Carbon Analyser (Shimadzu, UK). Non-purgeable organic carbon (NPOC) was measured after the acidification of samples with 9N sulfuric acid and catalytic combustion at 720°C as CO_2_. TN was measured as NO by chemiluminescence. The LoD was 18.3 μmol L^−1^ (TOC) and 30 μmol L^−1^ (TN), precision was ±2.0% as determined by comparison with diluted 41.7 mmol L^−1^ TOC certified stock standards to a concentration of 8.3 mmol L^−1^ and ±1.7% by comparison with diluted 14.3 mmol L^−1^ TN certified stock standards to a concentration of 3.6 mmol L^−1^ (Sigma TraceCERT^®^).

### Cellular stoichiometry

Glacier algal cellular carbon (C) and nitrogen (N) contents were determined for *n* = 5 samples from Morteratsch glacier for each location and time point. For this, GF/A filters were freeze-dried for 24 h to remove water, re-weighed and wrapped in individual 16 mm tin disks prior to elemental analysis using a Vario PYRO cube^®^ (Elementar, Stockport, UK). The detection limits of elemental concentrations were 0.001% for both elements measured, and the coefficient of variation (CV) for C and N according to 12 replicates of an organic analytical standard (NC Soil Standard 338 40025, cert. 341506, C = 2.31%, *N* = 0.23%; ThermoFisher Scientific, Bremen, Germany) were 1.76% and 1.13%, respectively. The molar content of carbon and nitrogen per sample was derived from the total recorded carbon and nitrogen area as follows:


$$\begin{array}{l} mMol\;=Ar_{x}\left(\frac{x\left[\%\right]}{100}\times w\right) \end{array}$$

where Ar_x_ is the relative atomic mass of nutrient x (e.g., carbon), *x* [%] is the derived percentage of nutrient x present in a processed sample, *w* is the total weight of the processed sample. Data are presented as molar C:N ratios.

### Data analysis

Analysis and plotting of data were completed using R v.4.2.1 (R Core Team, 2022). Each data series collected (cell abundance, biovolume, photophysiological, aqueous geochemistry, and stoichiometry) was tested for homogeneity of variance and normality of distribution. Statistical comparisons of photophysiology, aqueous geochemistry, and stoichiometry between sample locations and time points were investigated using one-way or two-way analysis of variance (ANOVA) with *post-hoc* Tukey Honest Significant Difference (HSD) analysis applied to all significant ANOVA results. ANOVA and Tukey HSD were also applied to comparisons between nutrient spiking treatments. To compare cell abundances and biovolumes between sampling locations and sampling times, Kruskal–Wallis rank sum tests (for non-parametric datasets) were employed. Linear regression analysis was employed to investigate potential relationships between cell abundance or biovolume and sample site slope. Aspect of the slope was treated as circular data (Von Mises distribution) and tested for correlation with cell abundance or biovolume using the circular correlation coefficient equivalent for Pearson's test for correlation (Fisher and Lee, 1983; Rao Jammalamadaka and Ramakrishna Sarma, 1988). Data were plotted using the ggplot2 package v.3.4.4.

## Results and discussion

A substantial glacier algal bloom was apparent during August 2022 at Morteratsch glacier, Switzerland (Figure 1), producing a conspicuous discoloration of the glacier's surface ice, particularly toward the terminus (Figure 2A). Sampling throughout the diurnal cycle across three elevations on the glacier allowed a broad characterization of the bloom, highlighting compositional and spatiotemporal heterogeneities in glacier algal biomass across the ice surface. These demonstrated how bloom magnitude does not scale linearly with melt season magnitude but rather upper limits on bloom carrying capacity may be determined by meltwater-driven redistribution and eventual export of glacier algal cells from the glacier.

### Cell abundance does not scale linearly with meltwater availability between seasons

**Figure 3:**
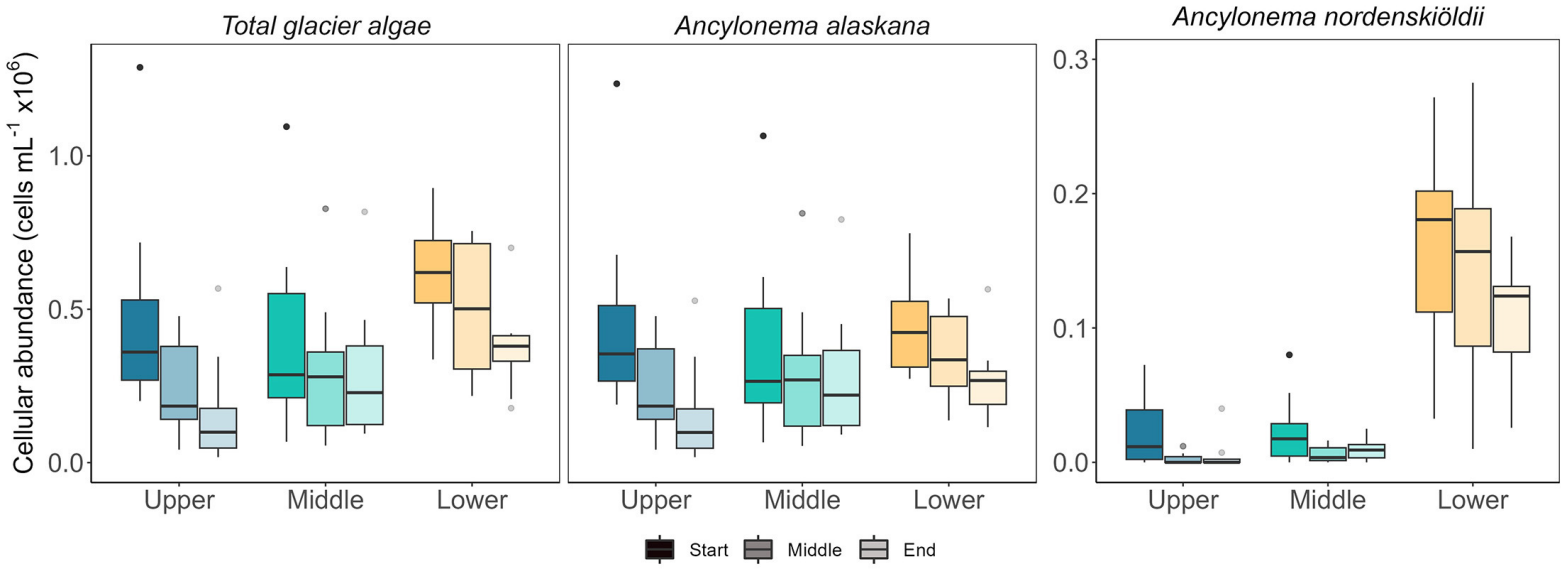
Cell abundance of glacier algae from ice surface samples across the sampling areas. Sub samples of melted ice surface samples were homogenized, and cells were counted to compare abundances ml^−1^. Boxplots show median, interquartile range, and minimum to maximum values with points showing potential outliers (*N* = 10), shading denotes the sampling period. **Left:** the total glacier algae cellular abundance. **Middle** and **right:** cellular abundance of *A. nordenskiöldii* and *A. alaskana*, respectively.

**Figure 4:**
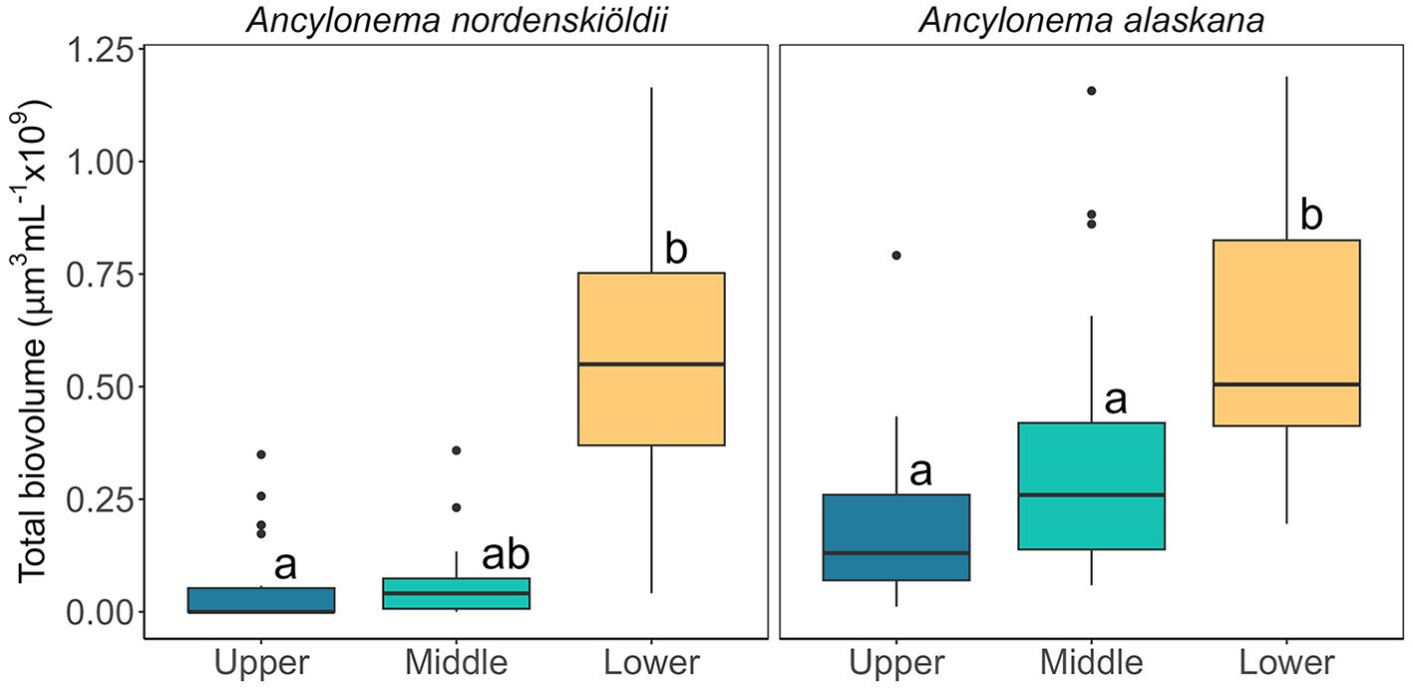
Total biovolume (μm^3^ ml^−1^) of the two glacier algal species present as determined by the average biovolume per cell per species and cell abundance. Larger cells as well as higher cell abundance was concentrated at the lower part of the glacier, resulting in a significantly larger biovolume in the samples collected from the lower site area. Boxplots show median, interquartile range, and minimum to maximum values, with points showing potential outliers (*N* = 15). Lower case letters indicate homogenous subsets determined through a Wilcoxon test in relation to location as the data are non-parametric, time was determined as not significantly different. **Left:** Total biovolume of *A. nordenskiöldii* (V = 2,415, *p* < 0.05). **Right:** Total biovolume of *A. alaskana* (V = 4095, *p* < 0.05).

Glacier algal communities were composed of both cosmopolitan glacier algal species at Morteratsch during 2022, with *Ancylonema alaskana* showing the greatest overall cellular abundance in all sampling locations as compared with *A. nordenskiöldii* (85% *A. alaskana* relative abundance vs. 15% *A. nordenskiöldii* across all data; Figure 3), though a more even split between species was apparent in our lowest sampling location based on total biovolume datasets (52% *A. alaskana* vs. 48% *A. nordenskiöldii*; Figure 4). These data are consistent with the study by Di Mauro et al. (2020) who reported an overall greater cellular abundance of *A. alaskana* relative to *A. nordenskiöldii* for the 2016 bloom in the same site; though contrasting findings based on DNA recovery. At present, we hold little-to-no knowledge on the factors that determine glacier algal community composition within or between sites and seasons; a key facet of bloom ecology yet to be understood. Indeed, it is likely that we do not yet have a full understanding of the true diversity of glacier algae across the cryosphere (Remias et al., 2023).

Glacier algal cellular abundance ranged from 1.78 × 10^4^ to 8.95 × 10^5^ cells ml^−1^ during August 2022 on Morteratsch glacier; at the higher end of abundances reported across the cryosphere during large bloom years (Stibal et al., 2017; Williamson et al., 2018; Di Mauro et al., 2020) but not substantially greater than previous records. For example, Di Mauro et al. (2020) reported an average of 2.03 × 10^4^ cells ml^−1^ from the same site in September 2016, during which melt may have been as little as a third of that in 2022 (World Glacier Monitoring Service, 2021, 2022). On the southwestern Greenland Ice Sheet (GrIS), known to harbor extensive glacier algal blooms, maximal abundances have been reported as 8.5 × 10^4^ cells ml^−1^ (Stibal et al., 2017), 1.6 × 10^4^ cells ml^−1^ (Williamson et al., 2018), and 3.0 × 10^5^ cells ml^−1^ at the margin (Yallop et al., 2012); comparable to our observations in Morteratsch. This suggests a more complex relationship between melt and glacier algal abundance and/or the presence of other controls on overall bloom carrying capacity.

### Spatiotemporal distribution of glacier algal cells

Spatiotemporal heterogeneity in glacier algal abundance recorded here indicated the potential melt-water-driven redistribution of (larger) glacier algal cells to down-glacier locations and presumptive export of biomass from the glacial system at Morteratsch; the latter a putative mechanism to set upper limits on bloom carrying capacity. At the diurnal scale, cellular abundance datasets recovered consistently higher abundances earlier in the day, when the volume of meltwater on the glacier surface was at its lowest (Figure 3). As melt increased throughout the day, corresponding estimates of abundance declined, reflecting dilution of assemblages by the higher melt fraction. The timing of sampling relative to the daily hydrograph is thus important for final estimates of abundance and should be considered in future sampling efforts.

Across our study locations, the abundance of *A. nordenskiöldii* was significantly higher in the lower region of the glacier (Figure 3), with a lower and more heterogenous abundance at mid and upper sites evidenced by a range of 0 cells ml^−1^ to 8.0 × 10^4^ cells ml^−1^ across samples. While the more abundant *A. alaskana* (cells ml^−1^) was more evenly distributed across the glacier (Figure 3), when cellular biovolume (μm^3^ cell^−1^) was incorporated into estimates, a clear spike in total biovolume (μm^3^ ml^−1^) was apparent at our lower site, highlighting the presence of larger *A. alaskana* and *A. nordenskiöldii* cells in this region of the glacier (Figure 4). Interestingly, this heterogeneity in glacier algal distribution was independent of surface ice slope or aspect. Data thus reflected our previous findings (Williamson et al., 2018), whereby true spatial trends in GrIS glacier algal blooms at the km scale were reflected only by total biovolume datasets rather than estimates of cellular abundance alone.

**Figure 5:**
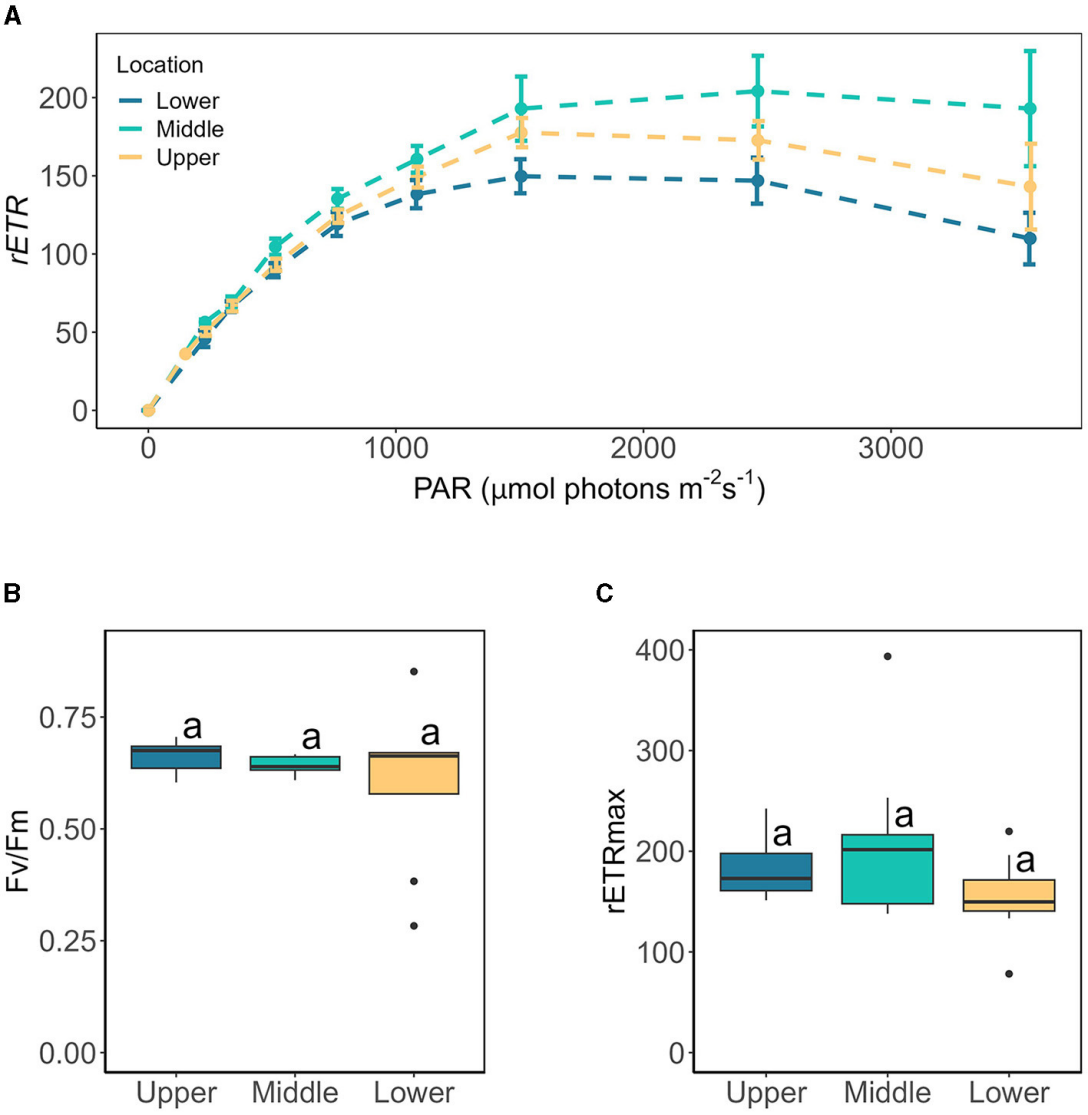
The photophysiology of glacier algae sampled from lower, middle, and upper glacier locations assessed by Rapid Light Curve (RLC) techniques, showing **(A)** rapid light curve traces of relative electron transport rates (mean ± SE, *N* = 10), **(B)** maximum quantum yield in the dark-adapted state (Fv/Fm) derived from the first RLC measurement in the dark, and **(C)** maximum electron transport rate (rETRmax) derived from Eilers and Peeters (1988) fitting of RLC rETR data relative to PAR. Boxplots show median, interquartile range, and minimum to maximum values, with points showing potential outliers (*N* = 10). Lowercase letters indicate homogenous subsets determined through a one-way ANOVA analysis of respective parameters in relation to location.

The presence of more and larger cells at our lowest sampling location irrespective of slope or aspect could reflect preferential growth within this region of the glacier and/or preferential transport of larger cells down glacier by meltwater, serving to concentrate assemblages at the terminus, with subsequent export to downstream environments. At larger spatial scales, the duration since snow-line retreat is a known factor controlling the progression of blooms, such that areas of glacial ice uncovered the earliest (i.e., at lower altitudes) provide more time for glacier algal blooms to develop (Tedstone et al., 2017; Williamson et al., 2018). This dynamic has been noted across the ablation zone of the GrIS (Tedstone et al., 2017; Williamson et al., 2018, 2020) as well as for valley glaciers (Takeuchi, 2013). However, sampling sites targeted here covered a relatively small altitudinal range (~100 meters of elevation gain) during a record melt year with rapid snow line retreat (GLAMOS, 2022; Cremona et al., 2023), and it is unlikely that substantial differences in exposure duration were experienced across our transect during 2022. PAM analysis did not indicate differences in community photophysiological state across our sampling locations (Figure 5), e.g., Fv/Fm remained consistently high across all locations at 0.66 ± 0.12 (Figure 5B). Additionally, aqueous geochemistry data and nutrient spiking incubations (see below) gave no indication of preferential conditions across our sampling locations.

Alternatively, larger cells (i.e., the filaments of *A. nordenskiöldii* and larger cells of *A. alaskana*) may have been preferentially transported over the weathering crust, concentrating in the lowest glacier sampling site. At the macro-scale, a clear concentration of glacier algal biomass was apparent at the glacier terminus throughout our sampling campaign (Figure 2). We also observed obvious down-glacier cell transport within meltwater flowing over the weathering crust via microscopic observations, indicating potential down-glacier concentration of cells (data not shown). Preferential movement of larger cells contrasts a previous report by Irvine-Fynn et al. (2021), which suggested that smaller cells are more likely to be transported through the weathering crust of the GrIS. However, it is likely that hydrological controls of cell distribution and weathering crust properties on steep valley glaciers differ from those of the flatter “dark zone” of the ice sheet; hence, more information is required on the meltwater-driven redistribution of glacier algal cells across the diversity of surface ice environments and its role in regulating bloom expansion vs. driving loss of cells from the system.

### Geochemistry of the heterogenous glacier algal bloom

**Figure 6:**
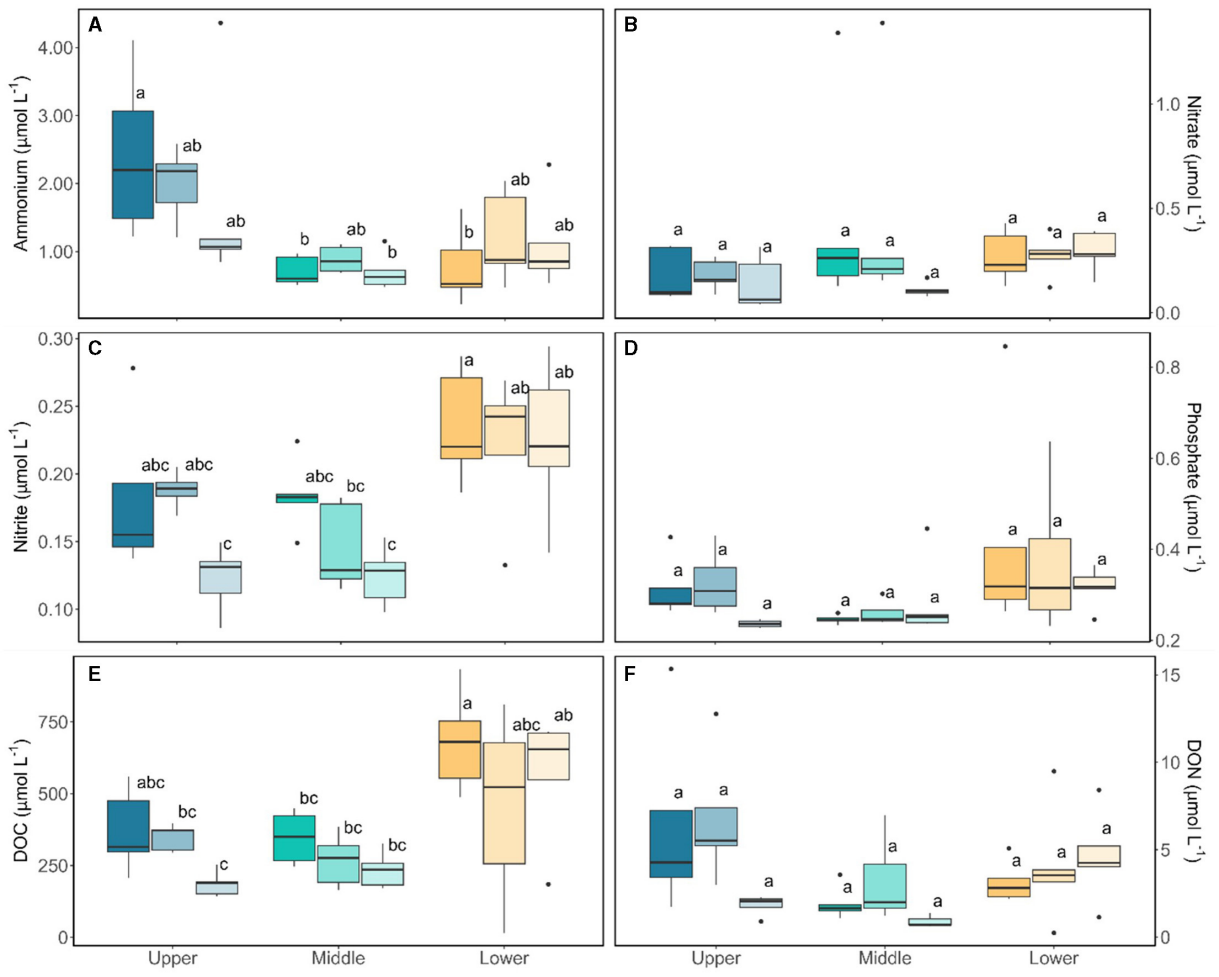
Aqueous geochemistry from the bulk samples taken from Morteratsch across locations and sampling period. Boxplots show median, interquartile range, and minimum to maximum values, with points showing potential outliers (*N* = 5) with shading denoting the sampling period. Lower case letters denote homogenous subsets determined through a two-way ANOVA analysis in relation to location and time. **(A)** Ammonium (F2,36 = 11.44, *p* < 0.05), **(B**) Nitrate, **(C)** Nitrite (F2,36 = 15.48, *p* < 0.05), **(D)** Phosphate, **(E)** Dissolved organic carbon (DOC) (F2,36 = 14.91, *p* < 0.05), **(F)** Dissolved organic nitrogen (DON).

**Figure 7:**
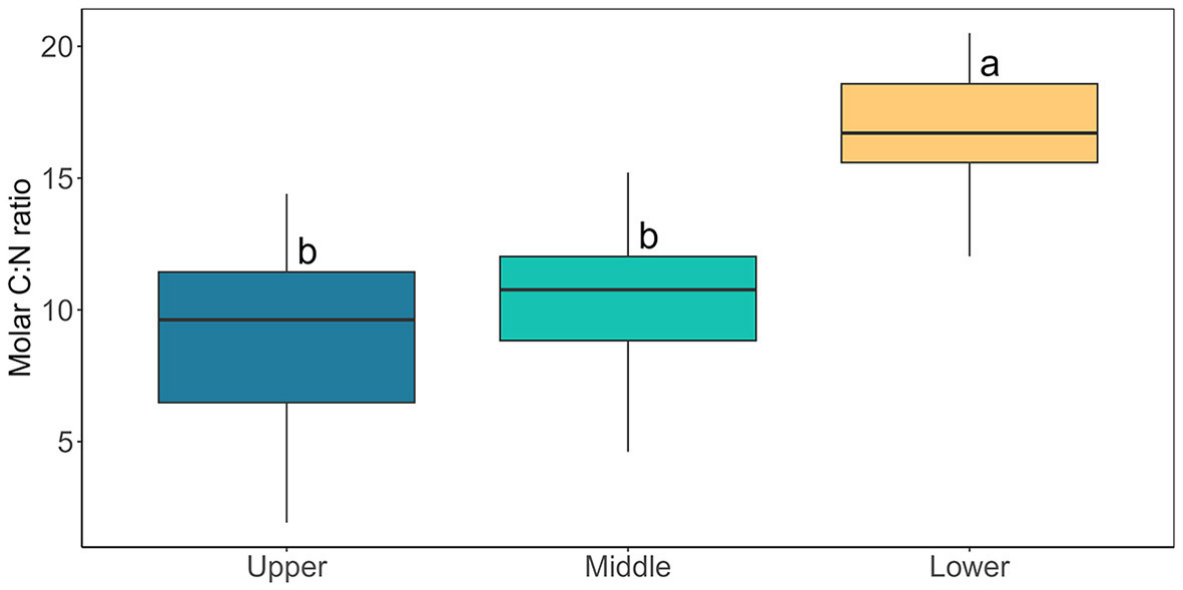
Cellular carbon to nitrogen ratio of bulk glacier algal samples collected from Morteratsch containing both *A. nordenskiöldii* and *A. alaskana*. Boxplots show median, interquartile range, and minimum to maximum values (*N* = 15). Lower case letters denote homogenous subsets determined through a one-way ANOVA analysis in relation to location (F2, 40 = 28.55, *p* < 0.05).

**Figure 8:**
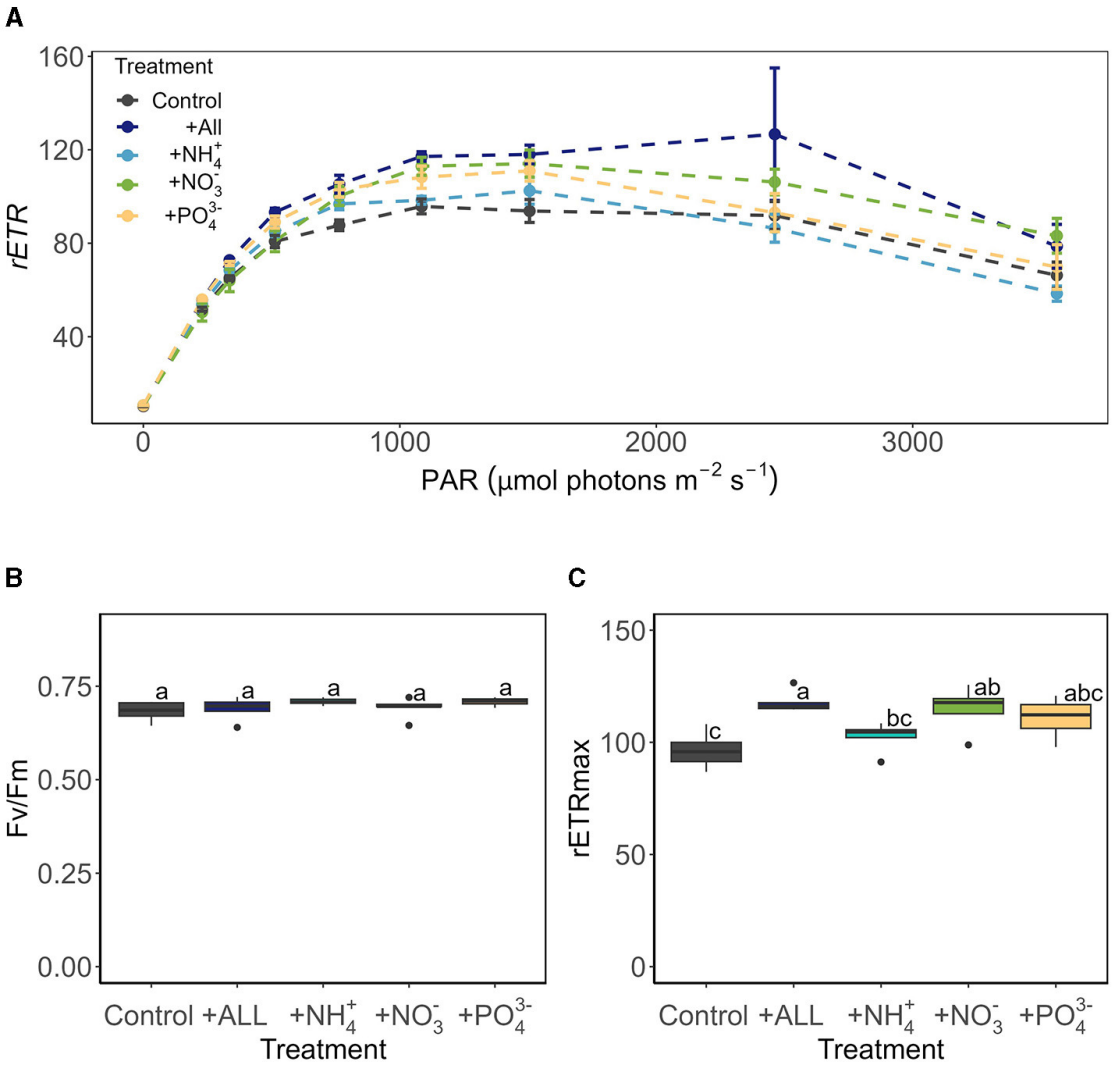
The photophysiology of glacier algae after 120 h of incubation under several nutrient treatments assessed by Rapid Light Curve (RLC) techniques, showing **(A)** rapid light curve traces of relative electron transport rates (mean ± SE, *N* = 5), **(B)** maximum quantum yield in the dark-adapted state (Fv/Fm) derived from the first RLC measurement in the dark, and **(C)** maximum electron transport rate (rETRmax) derived from Eilers and Peeters (1988) fitting of RLC rETR data relative to PAR (F5, 24 = 6.40, *p* < 0.05). Boxplots show median, interquartile range, and minimum to maximum values, with points showing potential outliers (*N* = 5). Lowercase letters indicate homogenous subsets determined through a one-way ANOVA analysis of respective parameters in relation to treatment.

Characterization of the bulk-phase aqueous geochemistry (Figure 6) once again demonstrated the paradox of highly abundant glacier algal communities thriving within oligotrophic surface ice (Williamson et al., 2018, 2020). Inorganic macro-nutrient concentrations ranged 0.22 – 4.36 μmol L^−1^ of ${\text{NH}}_{4}^{+}$, 0.04 – 1.38 μmol L^−1^ NO3^−^, 0.09 – 0.29 μmol L^−1^ ${\text{NO}}_{2}^{-}$, and 0.22 – 0.85 μmol L^−1^ ${\text{PO}}_{4}^{3-}$ (Figure 6), with a shift to dominance of organic speciation apparent by our sampling dates, i.e., 142.70 – 811.36 μmol L^−1^ dissolved organic carbon (DOC) and 0.23 – 15.34 μmol L^−1^ dissolved organic nitrogen (DON), reflecting known phase-shifts in the dominant geochemistry associated with blooms (Holland et al., 2019). Previously, Williamson et al. (2021) argued that glacier algae themselves may be adapted to low ambient nutrient concentrations through overall lower cellular N and P requirements. Indeed, we recorded similarly high C:N ratios here across sampling locations (average 12.28 ± 0.70 across all locations; Figure 7), with elevated C:N at our lowest sampling location corresponding to the presence of larger cells of both species; likely containing more carbon-rich phenolic pigmentation, rather than reflecting nutrient limitation at the terminus. To confirm this, algal communities sampled from our lower location were spiked with a series of nutrient additions (~10 times ambient ${\text{NH}}_{4}^{+}$, ${\text{NO}}_{3}^{-}$, and ${\text{PO}}_{4}^{3-}$ or a combination of all) and incubated for up to 120 h. Even after this 5-day duration, no clear indication of inorganic nutrient limitation was apparent for our sampled assemblages (Figure 8), with no difference across treatments in the maximum quantum yield in the dark-adapted state (Fv/Fm; Figure 8B) and minimal variance in rETRmax (Figure 8C).

These findings, taken together with previous studies (McCutcheon et al., 2021; Williamson et al., 2021), indicate that certainly at the point of sampling, glacier algae do not appear to be inorganic nutrient limited within surface ice environments of both mountain glaciers (here) or larger ice sheet systems (McCutcheon et al., 2021). This may reflect a true adaptation to low nutrient conditions (Williamson et al., 2021), efficient uptake and storage of those nutrients available within the system (Barcyté et al., 2019), and/or poor characterization of the available nutrients within the thin meltwater film that glacier algae reside. To date, researchers have typically employed the “bulk” method used here of ice sampling with subsequent melt and aqueous geochemistry characterization. This potentially dilutes the true nutrient available to glacier algae within their habitat, which may be enhanced due to ice solute exclusion and/or preferential elution of solute-rich fractions when glacier ice melts (Ginot et al., 2020; Holland et al., 2022). Thus, more work on true glacier algal nutrient requirements and nutrient availability within their habitat is needed to tackle the oligotrophic-bloom-paradox that is ubiquitous across the cryosphere.

## Conclusion

Characterization of a glacier algal bloom during a record melt year demonstrated how bloom proliferation does not scale linearly with meltwater availability on a steep valley glacier. Instead, a threshold appears to have been crossed, whereby meltwater shifts from being a promotor of glacier algal growth to a regulator of overall carrying capacity within surface ice via lateral transport of cells in surface meltwater. Abundances apparent during the record 2022 melt season at Morteratsch were high but not notably above previous estimates across the cryosphere. In contrast, spatiotemporal patterning in glacier algal biomass taken together with both macro- and micro-observations of bloom dynamics highlighted the potential for significant melt-water-driven, down-glacier transport of (larger) cells and presumptive export from the glacier, providing a first-order control on bloom carrying capacity. The findings were corroborated by aqueous geochemistry, algal eco-physiology, and nutrient spiking datasets that failed to reveal any biogeochemical rationale for the glacier algal distribution observed, once again highlighting the oligotrophic-bloom-paradox often reported across the cryosphere. Bottom-up controls on bloom proliferation will likely remain minimal as more of the glacial cryosphere becomes unlocked by climate warming in the future. However, our findings indicate that exponential proliferation of blooms as meltwater increases through time is likewise unlikely in valley glacier settings, where unknown thresholds of meltwater availability are surpassed and physical transport and export of cells from the system dominate. Many key facets of bloom ecology remain unknown as we head into the peak melt century.

## Data availability statement

The raw data supporting the conclusions of this article will be made available by the authors, without undue reservation.

## Author contributions

JM: Data curation, Formal analysis, Investigation, Methodology, Validation, Visualization, Writing—original draft, Writing—review & editing. EB: Data curation, Formal analysis, Investigation, Methodology, Software, Validation, Visualization, Writing—original draft, Writing—review & editing. ML: Investigation, Validation, Writing—review & editing. AB: Writing—review & editing. AT: Writing—review & editing. CW: Conceptualization, Funding acquisition, Methodology, Project administration, Resources, Supervision, Validation, Writing—original draft, Writing—review & editing.
